#  Genetic Cluster of Extended-Spectrum β-lactamase–Producing *Klebsiella pneumoniae* in Humans and Food, Switzerland, 2018–2019

**DOI:** 10.3201/eid3110.250253

**Published:** 2025-10

**Authors:** Lisandra Aguilar-Bultet, Elena Gómez-Sanz, Isabelle Vock, Monica Alt, Ruth Schindler, Laura Maurer Pekerman, Reto Furger, Lucas Eichenberger, Ingrid Steffen, Ana B. García-Martín, Tanja Stadler, Claudia Bagutti, Sarah Tschudin-Sutter

**Affiliations:** University Hospital Basel, University of Basel, Basel, Switzerland (L. Aguilar-Bultet, E. Gómez-Sanz, I. Vock, R. Schindler, L. Maurer Pekerman, A.B. García-Martín, S. Tschudin-Sutter); Institute of Veterinary Bacteriology, Vetsuisse Faculty, University of Bern, Bern, Switzerland (E. Gómez-Sanz); State Laboratory Basel-City, Basel (M. Alt, R. Furger, L. Eichenberger, C. Bagutti); Rothen Laboratory, Basel (I. Steffen); ETH Zurich, Basel (T. Stadler); Swiss Institute of Bioinformatics, Basel (T. Stadler)

**Keywords:** Klebsiella pneumoniae, extended-spectrum beta-lactamase, bacteria, antimicrobial resistance, food safety, sequence type 14, ST14, plasmids, Switzerland

## Abstract

We describe a cluster of genetically related extended-spectrum β-lactamase–producing *Klebsiella pneumoniae* sequence type 14 recovered from human clinical samples and an alfalfa-cress sample collected by systematic food sampling in Basel, Switzerland. Our findings suggest food could represent a reservoir contributing to spread of extended-spectrum β-lactamase–producing Enterobacterales.

Since 2017, the World Health Organization has classified extended-spectrum β-lactamase (ESBL)–producing *Klebsiella pneumoniae* as a critical public health priority (*1*). Healthcare settings are one known reservoir (*2*), but food might represent an underestimated and insufficiently investigated source (*3*–*5*). We investigated ESBL-producing Enterobacterales (ESBL-PE) in humans and the environment in Basel, Switzerland. 

## The Study

We designed and conducted a prospective study during June 2017–June 2019 to investigate the epidemiology of ESBL-PE in Basel, Switzerland (*6*) (Appendix). We collected monthly food and wastewater samples at predefined locations across Basel (*7*,*8*). The University Hospital Basel (USB) collected patient samples during routine clinical care. Microsynth AG (Balgach, Switzerland; https://www.microsynth.com) performed whole-genome sequencing of ESBL-PE isolates from clinical and food samples by using NextSeq 500 or 550 platforms (Illumina, https://www.illumina.com) on 150-bp paired-end reads, according to the manufacturer’s protocols (Appendix). We used core-genome multilocus sequence typing (cgMLST) to determine genetic relatedness among sequences. We used the MinION Mk1B or GridION sequencer and R.10.4.1 flow cells (all Oxford Nanopore Technologies, https://nanoporetech.com) to conduct nanopore sequencing on selected isolates from genetically related clusters (Appendix). 

We screened genomes from ESBL-PE isolates for antimicrobial resistance and plasmid genes. We conducted genomic comparisons against comprehensive international databases. 

**Figure 1:**
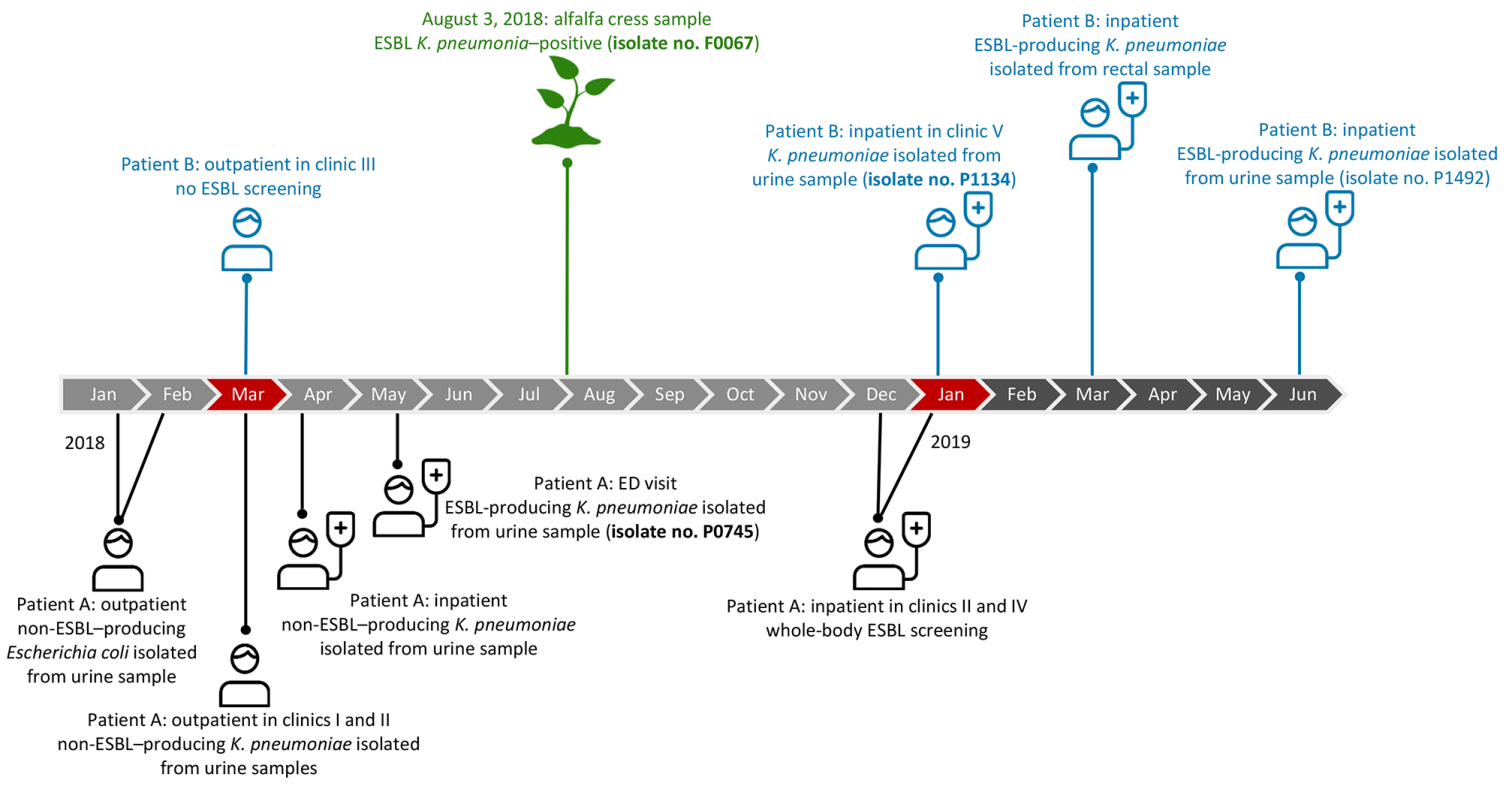
Timeline showing history of isolate collection in detection of ESBL-producing *Klebsiella pneumoniae* in human and food samples, Switzerland, 2018–2019. Bold font indicates isolates in the Basel cluster. Clinical ESBL-producing *K. pneumoniae* sequence type 14 isolates were collected from patient A (isolate no. P0745) in May 2018 and from patient B (isolate no. P1134) in January 2019. Both patients were treated at the University Hospital Basel (Basel, Switzerland) but at different times and in different clinics (Appendix). Isolates P1134 and P0745 formed a mixed-cluster of ESBL-producing *K. pneumoniae* isolates with food isolate (F0067), which was collected from alfalfa cress in August 2018. Months with overlapping visits or stays between both patients are depicted in red, and reference numbers of different clinics are provided to assess spatial proximity. ED, emergency department; ESBL, extended-spectrum β-lactamase.

During the 13-month study period, 12 of 947 food isolates tested positive for *K. pneumoniae* and had ESBL subsequently confirmed through phenotyping. The first clinical ESBL-producing *K. pneumoniae* sequence type (ST) 14 isolate (P0745) was recovered in May 2018 from a urine sample collected from a female outpatient (patient A) in her 70s treated in the USB emergency department for abdominal pain and general weakness (Figure 1). She had a history of recurrent urinary tract infections. At that emergency department visit, catheter-associated urinary tract infection was diagnosed and treated with ertapenem for 14 days after ESBL-producing *K. pneumoniae* was confirmed (Appendix).

Another clinical ESBL-producing *K. pneumoniae* ST14 isolate (P1134) was collected in January 2019 from a urine sample of another female patient (patient B) in her 70s admitted to USB for a cardiac disorder (Figure 1). Isolate P1134 was detected on day 6 of hospitalization and assessed as colonization. We investigated possible direct or indirect contact between the 2 patients but found no epidemiologic link (Appendix).

Phylogenetic analysis revealed an ESBL-producing *K. pneumoniae* ST14 isolate from 1 food sample that clustered with samples from the 2 patients. The food isolate (F0067) was recovered from 1 alfalfa cress sample organically produced in Switzerland and purchased from a Basel supermarket on August 3, 2018 (Figure 1). We identified 2 additional ESBL-producing *K. pneumoniae* isolates from samples collected from the same supermarket, 1 from mung beans purchased on November 9, 2018, and 1 from alfalfa cress purchased on February 8, 2019. All food samples were produced and packaged on the same farm in Switzerland. The 2 additional food isolates belonged to unrelated STs (the mung bean isolate was ST1310 and the second alfalfa cress isolate was ST2670), exhibited >1,990 allelic differences from F0067, and carried different combinations of ESBL genes and plasmid replicons (Appendix).

Genome comparison of isolates P0745, P1134, and F0067 revealed 6 cgMLST allelic differences and 12 single-nucleotide polymorphisms (SNPs) between F0067 and the clinical isolate of patient A (P0745). We also detected 3 cgMLST allelic differences and 7 SNPs difference between F0067 and the isolate from patient B (P1134). SNPs were scattered along the chromosomes, but none were in plasmids (Appendix Table 1).

**Figure 2:**
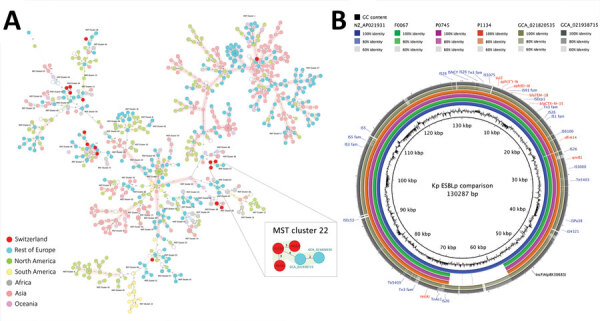
Phylogeny and related plasmids detected in ESBL–producing *Klebsiella pneumoniae* in human and food samples, Switzerland, 2018–2019. A) MST of 972 *Klebsiella pneumoniae* sequence type 14 international samples based on core-genome multilocus sequence typing (cgMLST) profiles (*K. pneumoniae* sensu lato cgMLST version 1.0 in SeqSphere+ version 9.0.0 [Ridom, https://www.ridom.de]). Inset image shows detail the mixed MST cluster 22. Color-filled circles denote the different geographic origin. Isolates of the same cluster (<15 allelic differences) are surrounded by a colored shadow. Numbers in connecting lines represent allelic distance values. B) Circular BLAST Ring Image Generator (BRIG, https://github.com/happykhan/BRIG) comparison of the ESBL plasmids from isolates in this study. Plasmid pWP2-W18-ESBL-06_2 (accession no. NZ_AP021931; blue ring) was identified as the closest hit in the PLSDB database version 2023_11_03_v2 (Universität des Saarlands, https://ccb-microbe.cs.uni-saarland.de/plsdb2025). Plasmid of the food isolate (F0067) ESBL-producing *K. pneumoniae* shown in green. Patient A isolate (P0745) shown in pink ring; patient B isolate (P1134) shown in orange ring. Brown-gray rings show short-read assembly from isolates GCA_21938715 and GCA_021820535 from the University Hospital Jena, Jena, Germany. Color intensity of concentric rings represents percent identity to the reference plasmid, pWP2-W18-ESBL-06_2. GC content and plasmid length are displayed as inner rings. Outer rings show antimicrobial resistance genes (red font), replicons (black font), and IS and Tn (blue font) sequences. ESBL, extended-spectrum β-lactamase; IS, insertion sequence; MST, minimum spanning tree; Tn, transposon.

cgMLST comparison of 967 global publicly available *K. pneumoniae* ST14 genomes and 14 isolates from our collection (including the clustered isolates) revealed 2 additional clinical isolates grouped within our cluster. Those isolates, collected in 2019 and 2020 at Jena University Hospital (Jena, Germany), showed 4–9 allelic differences from the isolates from Switzerland (Appendix Figure 2, panel A). The cluster reported in this study differed from other *K. pneumoniae* ST14 genomes by >58 alleles, and other isolates from Switzerland were dispersed across the minimum spanning tree, clustering with isolates from different countries and continents (Figure 2, panel A). The clustered isolates, including those from Germany, belonged to the same major lineage, indicating close phylogenetic relatedness (Appendix). Differences occurred only at the most specific level (10th node), suggesting minimal divergence within a clonal cluster.

The minimum mean average nucleotide identity among the 3 genomes from Basel was 99.99% (Appendix Figure 2, panel B). All isolates carried the KL2 capsule locus and the O1v2 locus, but we detected no additional known virulence genes. However, we detected a variant of the ESBL gene *bla*_SHV-106_, along with *oqxA*, *oqxB*, and *fosA6*, in those chromosomes (Appendix Table 2). The 3 isolates from Basel also shared a 120,635-bp ESBL plasmid of the IncFIA replicon type (Figure 2, panel B; Appendix Table 3), containing the ESBL gene *bla*_CTX-M-15_, and additional resistance genes for β-lactams (*bla*_TEM-1B_), sulfonamides (*sul2*), trimethoprim (*dfrA14*), aminoglycosides (*aph*(6)*-Id*, *aph*(*3′′*)*-Ib*), quinolones (*qnrB1*), and tetracycline (*tet*A) (Appendix Table 4). In addition, they shared a 193-kbp putative conjugative heavy metal resistance plasmid harboring genes for resistance to copper, arsenic, and silver (Appendix Table 3, Figure 3). We sequenced 1 additional *K. pneumoniae* ST14 isolate from patient B that shared most of those resistance genes and plasmid replicons, suggesting existence of a similar ESBL plasmid (Appendix Figure 1).

International comparison revealed plasmids from Basel had closest similarity (0.995 Mash identity [https://github.com/marbl/mash]) with a 130-kbp ESBL plasmid from a *K. pneumoniae* ST4 isolate collected in 2019 from wastewater in Japan (*9*) (Figure 2, panel B). That plasmid showed high synteny and included all antimicrobial resistance genes detected in the plasmids of the isolates from Switzerland and Germany (Appendix Table 4, Figure 4). However, the metal-resistant plasmid found in the isolates from Switzerland was absent from the isolates from Japan and Germany.

## Conclusions

We describe a cluster of closely related ESBL-producing *K. pneumoniae* ST14 isolates from human clinical samples collected at 2 hospitals in Europe and an alfalfa cress sample collected during systematic food sampling. Although we could not confirm a direct link between the patients and the food source, the close genetic relatedness and the geographic proximity and sampling dates suggest a recent common ancestor. Those findings highlight that food could be a potential reservoir in the spread of ESBL-PE. The national antimicrobial resistance surveillance system in Switzerland indicated stable resistance trends in *E. coli* and *K. pneumoniae* over the past decade (https://www.anresis.ch/antibiotic-resistance/resistance-data-human-medicine), supporting the relevance of our findings despite the time lapse after sample collection.

ESBL-producing *K. pneumoniae* clonal group 14, particularly CTX-M-15 producers, have caused human outbreaks globally (*10*,*11*) and have been detected in various fruits and vegetables (*3*), wastewater treatment plants, and rivers (*12*). The broad ecological range demonstrates that clonal group’s adaptability and its potential to disseminate antimicrobial resistance genes among isolates from food, environmental, and human sources. The close relatedness of strains and plasmids from our study to others identified globally further supports that potential. The detection of a similar ESBL plasmid in a wastewater *K. pneumoniae* ST4 isolate from Japan suggests environmental dissemination among diverse genetic lineages. In addition, a plasmid with metal tolerance and resistance genes might enable those bacteria to persist in harsh environmental conditions. That case, combined with an ESBL*-*producing *K. pneumoniae* food–human cluster we reported previously (*13*), adds to the limited evidence that food might be an overlooked and underestimated source of clinically relevant ESBL-producing *K. pneumoniae* clones (*4*).

In conclusion, this study highlights the value of investigating foodborne transmission routes to control the spread of clinically relevant antimicrobial-resistant bacterial strains. Stricter hygiene practices might be required throughout the food production, handling, and consumption cycle to prevent further dissemination of ESBL-PE to the general population. 

Appendix Additional information on detection of extended-spectrum β-lactamase–producing *Klebsiella pneumoniae* in human and food samples, Switzerland, 2018–2019.

## References

[1] World Health Organization. WHO bacterial priority pathogens list, 2024: bacterial pathogens of public health importance to guide research, development and strategies to prevent and control antimicrobial resistance. Geneva: The Organization; 2024.

[2] Vock I, Tschudin-Sutter S. Persisting intrahospital transmission of multidrug-resistant *Klebsiella pneumoniae* and challenges for infection control. Infect Control Hosp Epidemiol. 2019;40:904–9. 10.1017/ice.2019.153 31184564

[3] Mesbah Zekar F, Granier SA, Touati A, Millemann Y. Occurrence of third-generation cephalosporins–resistant *Klebsiella pneumoniae* in fresh fruits and vegetables purchased at markets in Algeria. Microb Drug Resist. 2020;26:353–9. 10.1089/mdr.2019.0249 31603740

[4] Silva-Bea S, Romero M, Parga A, Fernández J, Mora A, Otero A. Comparative analysis of multidrug-resistant *Klebsiella pneumoniae* strains of food and human origin reveals overlapping populations. Int J Food Microbiol. 2024;413:110605. 10.1016/j.ijfoodmicro.2024.110605 38308879

[5] Navon-Venezia S, Kondratyeva K, Carattoli A. *Klebsiella pneumoniae*: a major worldwide source and shuttle for antibiotic resistance. FEMS Microbiol Rev. 2017;41:252–75. 10.1093/femsre/fux013 28521338

[6] Stadler T, Meinel D, Aguilar-Bultet L, Huisman JS, Schindler R, Egli A, et al. Transmission of ESBL-producing Enterobacteriaceae and their mobile genetic elements-identification of sources by whole genome sequencing: study protocol for an observational study in Switzerland. BMJ Open. 2018;8:e021823. 10.1136/bmjopen-2018-021823 29455172 PMC5855333

[7] Gómez-Sanz E, Bagutti C, Roth JA, Alt Hug M, García-Martín AB, Maurer Pekerman L, et al. Spatiotemporal dissemination of ESBL-producing Enterobacterales in municipal sewer systems: a prospective, longitudinal study in the city of Basel, Switzerland. Front Microbiol. 2023;14:1174336. 10.3389/fmicb.2023.1174336 37250050 PMC10213686

[8] Gómez-Sanz E, Bagutti C, García-Martín AB, Roth JA, Alt Hug M, Maurer Pekerman L, et al. Extended-spectrum β-lactamase-producing Enterobacterales in diverse foodstuffs: a prospective, longitudinal study in the city of Basel, Switzerland. Front Microbiol. 2023;14:1295037. 10.3389/fmicb.2023.1295037 38075908 PMC10703160

[9] Sekizuka T, Tanaka R, Hashino M, Yatsu K, Kuroda M. Comprehensive genome and plasmidome analysis of antimicrobial resistant bacteria in wastewater treatment plant effluent of Tokyo. Antibiotics (Basel). 2022;11:1283. 10.3390/antibiotics11101283 36289941 PMC9598598

[10] Rodrigues C, Lanza VF, Peixe L, Coque TM, Novais Â. Phylogenomics of globally spread clonal groups 14 and 15 of *Klebsiella pneumoniae.* Microbiol Spectr. 2023;11:e0339522. 10.1128/spectrum.03395-22 37098951 PMC10269502

[11] Mshana SE, Hain T, Domann E, Lyamuya EF, Chakraborty T, Imirzalioglu C. Predominance of *Klebsiella pneumoniae* ST14 carrying CTX-M-15 causing neonatal sepsis in Tanzania. BMC Infect Dis. 2013;13:466. 10.1186/1471-2334-13-466 24099282 PMC3851032

[12] Martak D, Guther J, Verschuuren TD, Valot B, Conzelmann N, Bunk S, et al.; MODERN WP3 study group. Populations of extended-spectrum β-lactamase-producing *Escherichia coli* and *Klebsiella pneumoniae* are different in human-polluted environment and food items: a multicentre European study. Clin Microbiol Infect. 2022;28:447.e7–14. 10.1016/j.cmi.2021.07.022 34325070

[13] Aguilar-Bultet L, Bagutti C, Egli A, Alt M, Maurer Pekerman L, Schindler R, et al. Identification of a cluster of extended-spectrum Beta-lactamase-producing *Klebsiella pneumoniae* sequence type 101 isolated from food and humans. Clin Infect Dis. 2021;73:332–5. 10.1093/cid/ciaa1164 32776135 PMC8282321

