# Supplementary material for: Genetic Cluster of Extended-Spectrum β-lactamase–Producing Klebsiella pneumoniae in Humans and Food, Switzerland, 2018–2019
Source: Emerg Infect Dis. 2025 Oct;31(10):2017–21. doi: 10.3201/eid3110.250253 (PMC12483024; doi:10.3201/eid3110.250253)
Supplement: Appendix — Additional information on detection of extended-spectrum β-lactamase–producing Klebsiella pneumoniae in human and food samples, Switzerland, 2018–2019. [file 25-0253-Techapp-s1.pdf]

Article DOI: https://doi.org/10.3201/eid3110.250253

EID cannot ensure accessibility for supplementary materials supplied by authors. Readers who have difficulty accessing supplementary content should contact the authors for assistance.

Genetic Cluster of Extended-Spectrum β-lactamase–Producing *Klebsiella pneumoniae* in Humans and Food, Switzerland, 2018–2019

Appendix

Methodology

Prospective sampling and bacterial species identification

During the sampling period, June 2017 to June 2019, ESBL-PE specimens recovered from routine clinical practice were stored in a collection. At our institution, the following patient populations were systematically screened for ESBL carriage: all patients hospitalized in the same room as a patient colonized or infected with an ESBL-producing Enterobacterales for at least 24 hours; all patients admitted to the intensive care units and requiring mechanical ventilation; all patients transferred directly from a hospital abroad and all admitted asylum seekers.

Clinical isolates of ESBL-PE were obtained by standard culturing and in chromogenic screening agar (chrom ID ESBL, bioMérieux, Marcy-l'Étoile, France). Species identification was performed by Matrix-assisted laser desorption/ionization time-of-flight mass spectrometry (MALDI-TOF MS, Bruker Daltonics, Bremen, Germany), or by Vitek 2 System (BioMérieux, Marcy-l'Etoile, France).

Wastewater and foodstuff samples were also collected during the same period in the city of Basel, Switzerland. Sampling approach, isolation procedure and ESBL-PE species identification of wastewater and foodstuffs samples were detailed before (*1*,*2*). Chicken and green (herbs, salad, sprouts, vegetables) samples over a 2-year period (June 2017–June 2019). These samples were obtained from both large supermarket chains and small local food

retailers, covering all ten postcode areas of the City of Basel, Switzerland, as well as from the kitchen of the University Hospital Basel.

Antibiotic susceptibility testing

The Vitek 2 System was used for susceptibility testing of the clinical isolates. Confirmatory test for ESBL production was done with Etest® strips (BioMérieux, Marcy-l'Etoile, France) or with ROSCO disks (ROSCO, Taastrup, Denmark) using cefotaxime, ceftazidime or cefepime, each tested with and without clavulanic acid. Minimum inhibitory concentration (MIC) breakpoints were interpreted according to EUCAST guidelines (www.eucast.org).

Susceptibility testing of the food isolate reported here was performed using Vitek 2 compact (card N283) for all antibiotics but amikacin (disk assay using BioRad disk) and tobramycin (disk assay using ROSCO disk).

DNA extraction and sequencing of isolates

For short-read whole-genome sequencing, bacterial isolates were plated on blood agar plates (Columbia agar + 5% sheep blood, bioMérieux, Marcy-l'Étoile, France) and incubated overnight (O/N) at 37°C. Bacteria were collected with a loop and resuspended in Phosphate-Buffered Saline (PBS). Bacterial cells were harvested by centrifugation at 5000 x g for 10 min. Total DNA extraction and purification were carried out in a QIAcube machine (QIAGEN, Hilden, Germany) or a similar robot using the QIAamp DNA mini kit (QIAGEN, Hilden, Germany), according to the recommendations of the manufacturers.

Whole-genome sequencing was performed on ESBL-PE from clinical samples (one isolate per species from each body site per patient per hospital stay) and foodstuffs using the Illumina® technology (https://www.illumina.com), on the NextSeq 500/550 platforms (150 bp paired-end reads) at Microsynth AG (Balgach, Switzerland), according to the manufacturer's protocols. Library preparation was done using Nextera XT protocols (Illumina®, San Diego, USA).

For long-read whole-genome sequencing, bacterial isolates were likewise grown O/N on blood agar plates and subsequently submitted to the SeqCenter (Pennsylvania, USA). Genomic DNA was extracted using the ZymoBIOMICS DNA Miniprep Kit (https://zymoresearch.eu/products/zymobiomics-dna-miniprep-kit). Briefly, samples were aseptically scraped from the agar and resuspended in 750 µl of ZymoBIOMICS lysis solution. Suspended cells were transferred into the ZR BashingBead Lysis Tubes and

mechanically lysed using the MP FastPrep-24 lysis system with 1 min of lysis at maximum speed and 5 min of rest for five cycles. Further steps were carried out according to the manufacturer's specifications. Library preparation was performed using the PCR-free Oxford Nanopore technologies Ligation Sequencing Kit SQK-NBD114.24 with the NEBNext® Companion Module E7180L. Sequencing was performed on an Oxford Nanopore MinION Mk1B sequencer or a GridION sequencer using R.10.4.1 flow cells (Oxford Nanopore Technologies, Oxford, UK). Run design used the 400 bp sequencing mode with a minimum read length of 200 bp. Guppy v6.5.7 (https://nanoporetech.com/document/Guppy-protocol) was used for basecalling, demultiplexing and adaptor removal.

Genome assembly and annotation

Quality control, filtering and trimming of Illumina raw data was done with Fastp v0.20.0 (*3*). De novo genome assembly was done with Shovill v 1.1.0 (https://github.com/tseemann/shovill) using SPAdes v3.14.1 as assembler (*4*). Contigs shorter than 500 bp were excluded from the analysis. For samples with additional long-read sequencing data, a hybrid assembly was done with Unicycler v0.5.0 (*5*).

Genome annotation was done using Bakta v1.9.1 (*6*). Identification of antimicrobial resistance genes and plasmid replicons was performed with Resfinder v4.5.0 (*7*) and Plasmidfinder v2.0.1 (*8*), respectively with at least 80% identity and coverage.

Genetic relatedness based on core genome Multi Locus Sequence Typing (cgMLST)

Core genome Multi Locus Sequence Typing (cgMLST) with Ridom Seqsphere+ v9.0.0 (Ridom; Münster, Germany) was used to assess genetic relatedness among isolates. The cgMLST *K. pneumoniae sensu lato* scheme, which includes 2358 chromosomal genes, was used in the mentioned software. Isolates with less than 15 allelic differences (default threshold value of the aforementioned scheme) were considered part of the same cluster. Additionally, the classical *K. pneumoniae* MLST scheme was run to assign the sequence types (STs).

Whole-genome similarity analyses

Single nucleotide polymorphism (SNP) analyses were performed with Snippy (https://github.com/tseemann/snippy). For this, pairwise comparisons were done by mapping the short-reads from the human isolates to the hybrid-assembled genome of a reference isolate (food isolate or the oldest isolate when only human isolates were compared). Default

SNP quality filtering parameters were used. Pairwise Average Nucleotide Identity (ANI) among the three genomes was calculated with FastANI v1.3 (*9*).

cgMLST comparison with an international dataset

All 62,726 entries of *K. pneumoniae* genomes available were downloaded from the Genome database (https://www.ncbi.nlm.nih.gov/genome) of the Nacional Center for Biotechnology Information (NCBI) accessed on February 21, 2024 via "Klebsiella pneumoniae"[orgn:__txid573]. All genomes were genotyped *in silico* using the mlst software v.2.23.0 (https://github.com/tseemann/mlst) against the PubMLST database (*10*), and genomes belonging to the *K. pneumoniae* ST involved in the mixed cluster were recovered.

A Minimum Spanning Tree (MST) was generated based on the cgMLST profiles of isolates belonging to the ST14: isolates involved in the mixed cluster (n = 4, three clinical and one food), selected genomes from the global ST screening (n = 976), as well as genomes from our internal collection derived from the prospective project (n = 12) (*11*), which belonged to the same ST as the mixed cluster isolates. Nineteen samples which did not follow the minimal percent of targets found were excluded from the MST.

Additionally, the genomes of the Swiss cluster together with the genomes from Jena isolates were analyzed with the 629-gene cgMLST scheme hosted at the BIGSdb database (https://bigsdb.pasteur.fr/klebsiella/cgmlst-lincodes), which allowed to assign Life Identification Numbers (LIN) codes. The LIN codes provide a stable nomenclature to classify strains which is not affected by the addition of new genomes (*12*).

Plasmid identification and plasmid global comparison

Resultant circular extrachromosomal contigs were queried against the PLSDB database v2023_11_03_v2, a comprehensive resource of bacterial plasmids (*13*). Assigned plasmids from the investigated isolates and their best hits in PLSDB (using Mash identity) were compared using BLAST Ring Image Generator (BRIG) (*14*). Missing coding sequences between the plasmids were inspected using clinker (https://pegaso.microbiologia.ull.es/clinker-server.php).

Contigs (short-read assemblies) of the isolates obtained from patients of the University Hospital Jena were downloaded from NCBI (accession numbers GCA_21938715 and GCA_021820535). Their sequences were mapped to the closed ESBL-plasmid of the Japanese and Swiss isolates using BRIG.

Virulence

Kleborate implemented in Pathogenwatch (https://pathogen.watch) was used to screen the genomes for known *Klebsiella* virulence loci, such as yersiniabactin (*ybt*), colibactin (*clb*) (*15*), salmochelin (*iro*), aerobactin (*iuc*) (*16*), hypermucoidy (*rmpA*, *rmpA2*), and prediction of K (capsule) (*17*) and O antigen (LPS) (*18*).

Accession number BioProject

Raw data, genome sequences and metadata associated with the samples are registered at NCBI under the BioProject PRJNA1163283.

Ethics

This study received approval from the local ethics committee (EKNZ-2017 00100) and is part of the registered NRP project "Transmission of ESBL-producing Enterobacteriaceae" (ClinicalTrials.gov Identifier: NCT03465683; https://clinicaltrials.gov/study/NCT03465683). The committee classified it as a quality control project, not human research; thus, informed written consent was not required. Isolate names used in this study were created only for research purposes and do not correspond to the original isolate names of the hospital records.

Results

Food sample

During the 24-month sampling period, a total of 947 food samples were collected. Among them, nine alfalfa sprout samples, including two alfalfa-cress sprout samples, were collected from three different retailers and had different geographic origins. Two of these samples yielded ESBL-producing *K. pneumoniae* (including isolate F0067 from ST14 and F0114 from ST2670). These two isolates harbor the same ESBL-gene ($bla_{CTX-M-15}$) and other resistance genes; however, the plasmid replicons and other plasmid elements were different (Appendix Figure 1). An additional *K. pneumoniae* isolate belonging to ST1310 was recovered from the same retailer; its antimicrobial resistance profile and plasmid replicons are different from F0067 and F0114.

Patient A: Case description and clinical history

• History of recurrent urinary tract infections (UTIs) over the last years, treated with multiple antibiotics.

• Within the 5 months before the collection of the ESBL *K. pneumoniae* isolate (P0745), multiple non-ESBL *E. coli* and non-ESBL *K. pneumoniae* were detected in urine sample during outpatient visits for UTI episodes (Figure 1).

• ESBL-*E. coli* and ESBL-*K. pneumoniae* were also detected in urine within 3 months before the collection of the P0745 isolate, but these isolates were not stored.

• Due to recurrent UTIs, treatments administered included: nitrofurantoin, ciprofloxacin, cotrimoxazole, amoxicillin/clavulanic acid, ceftriaxone and piperacillin/tazobactam.

• May 2018: Presented to emergency department with abdominal pain and weakness; diagnosed with catheter-associated UTI. Urine culture yielded ESBL-*K. pneumoniae* of ST14 (isolate P0745) detected in urine. Treatment with ertapenem for 14 days.

Patient B: case description and clinical history

• Two outpatient visits took place in the previous year; no ESBL screening was performed.

• January 2019: Hospitalized for emergency cardiac surgery. Received perioperative treatment with cefuroxime. On day 6 of hospitalization, ESBL-*K. pneumoniae* ST14 (isolate P1134) was detected in urine (assessed as colonization). No prior ESBL history of colonization or infection documented.

• In the following months, hospitalized due to various symptoms and suspected UTI, ultimately diagnosed with a respiratory infection. Treatment changed from piperacillin/tazobactam to amoxicillin/clavulanic acid. ESBL-*K. pneumoniae* (isolate not stored) only found in rectal swab despite multiple body sites screening (Figure 1).

• Subsequently hospitalized for surgical revision; ESBL-*K. pneumoniae* ST14 (isolate P1492) was detected in urine on admission. Treated with piperacillin/tazobactam for hospital-acquired pneumonia.

• No ESBL-producing bacteria were detected in follow-up screenings 1 year later.

Assessment of epidemiologic link between both patients and between the patients and food sample

• March 2018: Both patients had outpatient visits in the same hospital building, with their closest visits 1 day apart but on different floors and different clinics.

• January – March 2019: Both patients were hospitalized during an overlapping period in January 2019 but on different floors and wards. In March 2019, patient B was at the same ward and floor as Patient A 3 months earlier; however, transmission linked to this ward is unlikely as she was already colonized in January.

Community contact

Both patients lived in different areas on the outskirts of the city of Basel, different as well as from the location of the food sample retailer (postal code 4055). In addition, the food producer was located 90 km away in Canton Luzern.

Consequently, no direct epidemiologic link could be identified between both patients nor between the clinical and the food isolates.

Additional genome comparisons

Hybrid assembly revealed a circular chromosome of ≈5.3 Mb in the three isolates from Basel. Comparison between the isolates from patient A (P0745) and patient B (P1134) revealed three allelic differences (8 SNPs), while the second isolate from patient B (P1492) differed from P1134 by only three alleles.

The LIN codes identified reinforce that the samples are closely related. F0067, P1134 and GCA_021938715.1 showed identical LIN codes (0_0_1_1_35_0_0_0_0_0), while P0745 and GCA_021820535.1 showed a slightly different variation at the last position (0_0_1_1_35_0_0_0_0_2 and 0_0_1_1_35_0_0_0_0_1, respectively). They shared the same LIN codes with other entries in the database (scgST-8127, scgST-24156, scgST-21297 and scgST-24154); however, no metadata was accessible in BIGSdb for those genomes. When cross-checking in Pathogenwatch (https://pathogen.watch/), the metadata was identified and corresponded to the same samples previously submitted to the international databases.

Detailed plasmid sequence analyses

The 120,635 bp ESBL-plasmids present in the three isolates harbored a replication initiation protein (RepE), a plasmid partitioning system (ParA, ParB) and a resolvase-coding gene; a toxin-antitoxin replicon stabilization system (VapC toxin, VapB antitoxin), which may also contribute to plasmid maintenance; and numerous mobilization genes, including insertion sequences (ISs) and transposable elements. Overall, they have a notable lower GC content (50.63%; 49.74%) than that of their bacterial chromosome (57.5%).

International plasmid comparison using the PSLDB database revealed 29 hits. Closest similarity (length, content, Mash identity) corresponded to a circular ESBL-plasmid of

130,287 bp (0.995 Mash identity, accession number NZ_AP021931.1) of the replicon type IncFIA, from a *K. pneumoniae* ST4 recovered from wastewater in Japan in 2018 (Figure 2, panel B) (*19*). This ESBL-plasmid (pWP2-W18-ESBL-06_2) exhibited high synteny and covered all antimicrobial resistance genes detected in the mixed cluster (Appendix Table 4). The additional 10 kbp fragment exclusively present in this ESBL-plasmid contained genes involved in plasmid adaptation, *i.e.*: plasmid stability and segregation (*parM*) as well as an antirestriction system (*ardA*) involved in overcoming the host bacterial cell's restriction modification systems. Two additional 79.75 kbp circular plasmids from two *K. pneumoniae* ST15 isolates of human origin from Houston Methodist Hospital in the USA (pKPN1402_3 from bile, accession number CP128712.1; pKPN1409_3 from blood, accession number CP128707.1) revealed high identity (0.992 Mash identity in both cases) and synteny with those of our cluster isolates but lacked all antimicrobial resistance markers (data not shown). These two plasmids contained the unique region detected in pWP2-W18-ESBL-06_2.

BRIG comparison between contigs from Jena's isolates (downloaded from the NCBI Assembly database) and the closed ESBL-plasmids from pWP2-W18-ESBL-06, F0067, P1134 and P0745 isolates, showed that they not only resemble the plasmid from Swiss isolates, but also contained almost all elements of the 130 kb plasmid pWP2-W18-ESBL-06_2 (Figure 2, panel B), suggesting the presence of a similar 130 kb ESBL-plasmid. However, long-read sequencing data are needed to fully confirm the structure of the plasmid in Jena's isolates, as the available contigs are derived from short-read-based assembly.

All three clustered isolates shared a putative conjugative metal resistance plasmid of the replicon types IncFIB(K) and IncFII(pKP91). The plasmids from isolates F0067, P0745 and P1134, designated pKP14-F0067_1, pKP14-P0745_1 and pKP14-P1134_1, respectively, were 192,895 bp, with an average GC content of 52.09% (Appendix Table 3). Based on the presence of a 34 kb block of genes that constitute the core conjugative transfer system (*traI*, *traD*, *traA*, *traB*, *traC*, *traF*, *traG*, *traH*, *traJ*, *traK*, *traL*, *traM*, *traN*, *traP*, *traQ*, *traS*, *traT*, *traU*, *traV*, *traW*, *trbB*, *trbC*, *trbE*, *trbF*, *trbI*) it is highly likely that these plasmids are conjugative and thus more easily transmissible to other microorganisms. These plasmids harbored genes encoding for multicomponent transmembrane transporters involved in the efflux of copper, silver, arsenic, and iron (Appendix Figure 3), and several toxin-antitoxin systems, which may also contribute to plasmid maintenance. Additional ABC transporter components were detected, some of which appeared to be involved in urea transfer. Moreover, numerous mobilization genes (ISs, transposable elements) were present

throughout the plasmid genomes, with some flanking the metal tolerance/resistance transporters. Altogether, these elements may add the plasmid further adaptation properties. Two distantly located replication initiation protein-coding genes (*repB*, *repA*), and a plasmid partitioning system (*spo0J*, *sopA*) were detected as part of the plasmid core sequence.

International plasmid comparison using PSLDB revealed 25 hits. Closest similarity (length, content, Mash identity) corresponded to a circular plasmid of 195,131 bp (0.999 Mash identity), named pKqq_SB98_2 (accession number NZ_CP084805.1), with the replicon types IncFIB(K) and IncFII(pKP91), from an environmental sample from *Klebsiella quasipneumoniae* ST622 (*20*). Longest hits corresponded to two circular plasmid genomes of 208 and 202 kbp (0.992, 0.993 Mash identity, accession numbers NZ_CP070582.1 and NZ_CP065832.1, respectively) from *K. pneumoniae* isolates, originated from humans (sputum, urine, respectively) from Czech Republic and USA, respectively.

References

1. Gómez-Sanz E, Bagutti C, Roth JA, Alt Hug M, García-Martín AB, Maurer Pekerman L, et al. Spatiotemporal dissemination of ESBL-producing Enterobacterales in municipal sewer systems: a prospective, longitudinal study in the city of Basel, Switzerland. Front Microbiol. 2023;14:1174336. PubMed https://doi.org/10.3389/fmicb.2023.1174336

2. Gómez-Sanz E, Bagutti C, García-Martín AB, Roth JA, Alt Hug M, Maurer Pekerman L, et al. Extended-spectrum β-lactamase-producing Enterobacterales in diverse foodstuffs: a prospective, longitudinal study in the city of Basel, Switzerland. Front Microbiol. 2023;14:1295037. PubMed https://doi.org/10.3389/fmicb.2023.1295037

3. Chen S, Zhou Y, Chen Y, Gu J. fastp: an ultra-fast all-in-one FASTQ preprocessor. Bioinformatics. 2018;34:i884–90. PubMed https://doi.org/10.1093/bioinformatics/bty560

4. Bankevich A, Nurk S, Antipov D, Gurevich AA, Dvorkin M, Kulikov AS, et al. SPAdes: a new genome assembly algorithm and its applications to single-cell sequencing. J Comput Biol. 2012;19:455–77. PubMed https://doi.org/10.1089/cmb.2012.0021

5. Wick RR, Judd LM, Gorrie CL, Holt KE. Unicycler: resolving bacterial genome assemblies from short and long sequencing reads. PLOS Comput Biol. 2017;13:e1005595. PubMed https://doi.org/10.1371/journal.pcbi.1005595

6. Schwengers O, Jelonek L, Dieckmann MA, Beyvers S, Blom J, Goesmann A. Bakta: rapid and standardized annotation of bacterial genomes via alignment-free sequence identification. Microb Genom. 2021;7:000685. PubMed https://doi.org/10.1099/mgen.0.000685

7. Bortolaia V, Kaas RS, Ruppe E, Roberts MC, Schwarz S, Cattoir V, et al. ResFinder 4.0 for predictions of phenotypes from genotypes. J Antimicrob Chemother. 2020;75:3491–500. PubMed https://doi.org/10.1093/jac/dkaa345

8. Carattoli A, Zankari E, García-Fernández A, Voldby Larsen M, Lund O, Villa L, et al. In silico detection and typing of plasmids using PlasmidFinder and plasmid multilocus sequence typing. Antimicrob Agents Chemother. 2014;58:3895–903. PubMed https://doi.org/10.1128/AAC.02412-14

9. Jain C, Rodriguez-R LM, Phillippy AM, Konstantinidis KT, Aluru S. High throughput ANI analysis of 90K prokaryotic genomes reveals clear species boundaries. Nat Commun. 2018;9:5114. PubMed https://doi.org/10.1038/s41467-018-07641-9

10. Jolley KA, Maiden MC. BIGSdb: Scalable analysis of bacterial genome variation at the population level. BMC Bioinformatics. 2010;11:595. PubMed https://doi.org/10.1186/1471-2105-11-595

11. Stadler T, Meinel D, Aguilar-Bultet L, Huisman JS, Schindler R, Egli A, et al. Transmission of ESBL-producing Enterobacteriaceae and their mobile genetic elements-identification of sources by whole genome sequencing: study protocol for an observational study in Switzerland. BMJ Open. 2018;8:e021823. PubMed https://doi.org/10.1136/bmjopen-2018-021823

12. Hennart M, Guglielmini J, Bridel S, Maiden MCJ, Jolley KA, Criscuolo A, et al. A Dual Barcoding approach to bacterial strain nomenclature: genomic taxonomy of *Klebsiella pneumoniae* strains. Mol Biol Evol. 2022;39:msac135. PubMed https://doi.org/10.1093/molbev/msac135

13. Galata V, Fehlmann T, Backes C, Keller A. PLSDB: a resource of complete bacterial plasmids. Nucleic Acids Res. 2019;47(D1):D195–202. PubMed https://doi.org/10.1093/nar/gky1050

14. Alikhan NF, Petty NK, Ben Zakour NL, Beatson SA. BLAST Ring Image Generator (BRIG): simple prokaryote genome comparisons. BMC Genomics. 2011;12:402. PubMed https://doi.org/10.1186/1471-2164-12-402

15. Lam MMC, Wick RR, Wyres KL, Gorrie CL, Judd LM, Jenney AWJ, et al. Genetic diversity, mobilisation and spread of the yersiniabactin-encoding mobile element ICEKp in *Klebsiella pneumoniae* populations. Microb Genom. 2018;4:e000196. PubMed https://doi.org/10.1099/mgen.0.000196

16. Lam MMC, Wyres KL, Judd LM, Wick RR, Jenney A, Brisse S, et al. Tracking key virulence loci encoding aerobactin and salmochelin siderophore synthesis in *Klebsiella pneumoniae.* Genome Med. 2018;10:77. PubMed https://doi.org/10.1186/s13073-018-0587-5

17. Wyres KL, Wick RR, Gorrie C, Jenney A, Follador R, Thomson NR, et al. Identification of *Klebsiella* capsule synthesis loci from whole genome data. Microb Genom. 2016;2:e000102. PubMed https://doi.org/10.1099/mgen.0.000102

18. Wick RR, Heinz E, Holt KE, Wyres KL. Kaptive Web: user-friendly capsule and lipopolysaccharide serotype prediction for *Klebsiella* genomes. J Clin Microbiol. 2018;56:e00197-18. PubMed https://doi.org/10.1128/JCM.00197-18

19. Sekizuka T, Tanaka R, Hashino M, Yatsu K, Kuroda M. Comprehensive genome and plasmidome analysis of antimicrobial resistant bacteria in wastewater treatment plant effluent of Tokyo. Antibiotics (Basel). 2022;11:1283. PubMed https://doi.org/10.3390/antibiotics11101283

20. Hawkey J, Vezina B, Monk JM, Judd LM, Harshegyi T, López-Fernández S, et al. A curated collection of *Klebsiella* metabolic models reveals variable substrate usage and gene essentiality. Genome Res. 2022;32:1004–14. PubMed https://doi.org/10.1101/gr.276289.121

Appendix Table 1. Single nucleotide polymorphisms (SNPs) detected at whole-genome level comparisons taking the genome of the food isolate F0067 as reference

Query	Contig	Position	Reference nucleotide	Alternative nucleotide	Evidence	Feature type	Nucleotide position	Amino acid position	Effect	Gene	Product
P0745	Contig_1 (chrom.)	401316	T	A	A:85 T:0	CDS	1015/1188	339/395	missense_variant c.1015A>T p.Thr339Ser	*tsgA*	MFS transporter TsgA
		636410	G	T	T:90 G:0	CDS	75/1824	25/607	synonymous_variant c.75G>T p.Ala25Ala		diol dehydratase reactivase subunit α
		637096	G	C	C:75 G:0	CDS	761/1824	254/607	missense_variant c.761G>C p.Gly254Ala		diol dehydratase reactivase subunit α
		780263	A	G	G:112 A:0	CDS	1279/2874	427/957	missense_variant c.1279A>G p.Thr427Ala	*gcvP*	aminomethyl-transferring glycine dehydrogenase
		1231441	A	G	G:108 A:0	CDS	292/906	98/301	missense_variant c.292A>G p.Ser98Gly	*era*	GTPase Era
		1232457	C	T	T:82 C:0	CDS	391/738	131/245	synonymous_variant c.391C>T p.Leu131Leu	*recO*	DNA repair protein RecO
		2104875	T	C	C:79 T:0	CDS	270/465	90/154	synonymous_variant c.270T>C p.Thr90Thr	*nlpC*	Cell wall-associated hydrolase, NlpC_P60 family
		2502050	T	C	C:89 T:0	CDS	402/1143	134/380	synonymous_variant c.402A>G p.Lys134Lys	*pqqE*	pyrroloquinoline quinone biosynthesis protein PqqE
		2722790	A	T	T:105 A:0	CDS	56/1089	19/362	missense_variant c.56T>A p.Val19Glu		putative ATPase
		3136967	T	A	A:91 T:1						
		3376443	A	G	G:116 A:0						
		4338130	C	T	T:82 C:0						
P1134	Contig_1 (chrom.)	780263	A	G	G:130 A:0	CDS	1279/2874	427/957	missense_variant c.1279A>G p.Thr427Ala	*gcvP*	aminomethyl-transferring glycine dehydrogenase
		1231441	A	G	G:126 A:0	CDS	292/906	98/301	missense_variant c.292A>G p.Ser98Gly	*era*	GTPase Era
		2104875	T	C	C:82 T:0	CDS	270/465	90/154	synonymous_variant c.270T>C p.Thr90Thr	*nlpC*	Cell wall-associated hydrolase, NlpC_P60 family
		2722790	A	T	T:69 A:0	CDS	56/1089	19/362	missense_variant c.56T>A p.Val19Glu		putative ATPase
		3136967	T	A	A:95 T:1						
		3430652	G	A	A:16 G:1	CDS	1131/7518	377/2505	synonymous_variant c.1131C>T p.Ser377Ser	*bapA*	BapA prefix-like domain-containing protein
		4338130	C	T	T:81 C:0						

Appendix Table 2. Antimicrobial resistance profile of isolates involved in the mixed cluster and location of antimicrobial resistance genes detected.

Antibiotic class†	Antibiotic	P0745	F0067	P1134	Plasmid located AMR genes	Chromosome located AMR genes
Penicillins	Amoxicillin/clavuvanic acid	R	S	R	*bla*CTX-M-15 (ESBL), *bla*TEM-1B	*bla*SHV-106 (ESBL), *bla*SHV-28‡
Penicillins	Piperacillin/tazobactam	S	S	S		
BS Penicillins	Ampicillin/amoxicillin	R	R	R		
4GC	Cefepim	I	I	I		
3GC	Cefpodoxim	R	R	R		
3GC	Ceftazidim	R	R	R		
3GC	Ceftriaxon	R	R	R		
Carbapenems	Ertapenem	S	S	S		
	Imipenem	S	S	S		
	Meropenem	S	S	S		
Quinolons	Ciprofloxacin	R	I	R	*qnrB1*	*oqxAB*
	Levofloxacin	R	S	R		
	Norfloxacin	R	R	R		
Aminoglycocides	Tobramycin	S	S	S		
	Amikacin	S	S	S		
	Streptomycin	NT	NT	NT	*aph*(6)-Id, *aph*(3'')-Ib	
Polymyxins	Colistin	S	NT	S		
Sulfonamides	Cotrimoxazol	R	R	R	*dfrA14*, *sul2*	
Phosphonic	Fosfomycin/trometamol§	R	S	S		*fosA6*
Tetracyclines	Tetracycline	NT	NT	NT	*tet*(A)	

*I, intermediate or increased exposure; NT, not tested; R, resistant; S, sensitive.
†Antibiotic classes include the following: BS, broad spectrum; 3GC, 3rd generation cephalosporins; 4GC, 4th generation cephalosporins;
‡ Both *bla*SHV variants were identified at the same position and at equal confidence (99.88% identity, 100% coverage over 861 bp). Due to identical alignment results, the exact allele could not be unambiguously determined.
§ Fosfomycin alone for F0067.

Appendix Table 3. Plasmid types detected among the two plasmids present among the mixed cluster isolates.

Inc type	% Identity to reference (GenBank accession no.)	Query/template length	Isolate ID	Plasmid ID	Plasmid bp length*
IncFII(pKP91)	96.07 (CP000966)	229/230	F0067, P0745, P1134	pKP14-F0067_1, pKP14_P0745_1, pKP14_P1134_1	192,895
IncFIB(K)	98.75 (JN233704)	560/560	F0067, P0745, P1134	pKP14-F0067_1, pKP14_P0745_1, pKP14_P1134_1	192,895
IncFIA(pBK30683)	97.29 (KF954760)	295/295	F0067, P0745, P1134	pKP14-F0067_2, pKP14_P0745_2, pKP14_P1134_2	120,635

*Using nanopore assembly data.

Appendix Table 4. Overview of the ESBL-plasmids detected from the mixed-cluster isolates (pKP14-F0067_2, pKP14_P0745_2, pKP14_P1134_2) and the best plasmid hit from PLSDB (pWP2-W18-ESBL-06_2), and comparative details of the antimicrobial resistance genes identified.

Antibiotic class*	Antimicrobial resistance gene	% Identity/coverage to reference (GenBank accession no.)	Isolate ID	Plasmid ID	Plasmid bp length†
ESBL	*bla*$_{CTX-M-15}$	100/100 (AY044436)	F0067, P0745, P1134	pKP14-F0067_2, pKP14_P0745_2, pKP14_P1134_2	120,635
	bla$_{CTX-M-15}$	100/100 (AY044436)	WP2-W18-ESBL-06‡	pWP2-W18-ESBL-06_2	130,287
3GC	*bla*$_{TEM-1B}$	100/100 (AY458016)	F0067, P0745, P1134	pKP14-F0067_2, pKP14_P0745_2, pKP14_P1134_2	120,635
	bla$_{TEM-1B}$	100/100 (AY458016)	WP2-W18-ESBL-06	pWP2-W18-ESBL-06_2	130,287
Streptomycin	*aph (6)-Id*, *aph(3")-Ib*	100/100 (M28829, AF321551)	F0067, P0745, P1134	pKP14-F0067_2, pKP14_P0745_2, pKP14_P1134_2	120,635
	aph (6)-Id, *aph(3")-Ib*	100/100 (M28829, AF321551)	WP2-W18-ESBL-06	pWP2-W18-ESBL-06_2	130,287
Sulfonamides	*sul2*	100/100 (AY034138)	F0067, P0745, P1134	pKP14-F0067_2, pKP14_P0745_2, pKP14_P1134_2	120,635
	sul2	100/100 (AY034138)	WP2-W18-ESBL-06	pWP2-W18-ESBL-06_2	130,287
Trimethoprim	*dfrA14*	100/100 (KF921535)	F0067, P0745, P1134	pKP14-F0067_2, pKP14_P0745_2, pKP14_P1134_2	120,635
	dfrA14	100/100 (KF921535)	WP2-W18-ESBL-06	pWP2-W18-ESBL-06_2	130,287
Tetracyclines	*tet*(A)	100/100 (AJ517790)	F0067, P0745, P1134	pKP14-F0067_2, pKP14_P0745_2, pKP14_P1134_2	120,635
	tet(A)	100/100 (AJ517790)	WP2-W18-ESBL-06	pWP2-W18-ESBL-06_2	130,287
Fluoroquinolones	*qnrB1*	100/100 (DQ351241)	F0067, P0745, P1134	pKP14-F0067_2, pKP14_P0745_2, pKP14_P1134_2	120,635
	qnrB1	100/100 (DQ351241)	WP2-W18-ESBL-06	pWP2-W18-ESBL-06_2	130,287

*Antibiotic classes include the following: ESBL, extended-spectrum β-lactamase; 3GC, 3rd generation cephalosporins.
†Using nanopore assembly data.
‡Accession number NZ_AP021931.1. This isolate harbors 2 additional unrelated plasmids: pWP2-W18-ESBL-06_1 (140,912 bp) and pWP2-W18-ESBL-06_3 (3,981 bp) (*19*).

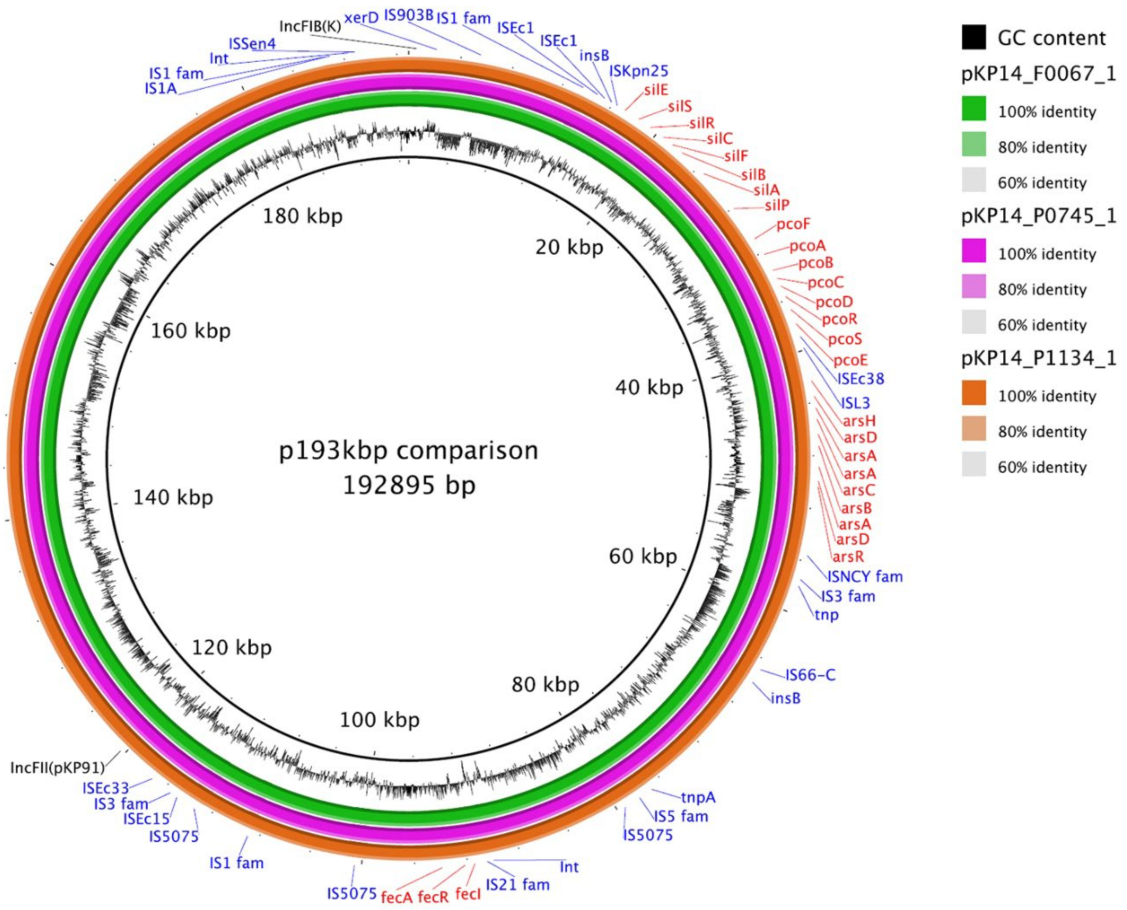

Appendix Figure 1. BRIG comparison of the ESBL-plasmids. Blue ring: plasmid pWP2-W18-ESBL-06_2 (NZ_AP021931) - best hit of the PLSDB database (v. 2023_11_03_v2). Green ring: ESBL-plasmid of the food ESBL-*K. pneumoniae* isolate (F0067). Pink ring: ESBL-plasmid of ESBL-*K. pneumoniae* isolate from patient A (P0745). Orange ring: ESBL-plasmid of the ESBL-*K. pneumoniae* isolate of patient B (P1134). Red ring: short-read assembly of the additional sequenced ESBL-*K. pneumoniae* isolate of Patient B (P1492). Brown-gray rings: short-read assembly from isolates GCA_21938715 and GCA_021820535 from the University Hospital Jena, Germany. Aqua ring: short-read assembly of the additional sequenced ESBL-*K. pneumoniae* food isolate F0114 (from ST2670). The color intensity of the concentric rings represents the percentage of identity compared to the reference used (pWP2-W18-ESBL-06_2). GC content and plasmid length are displayed as inner rings.

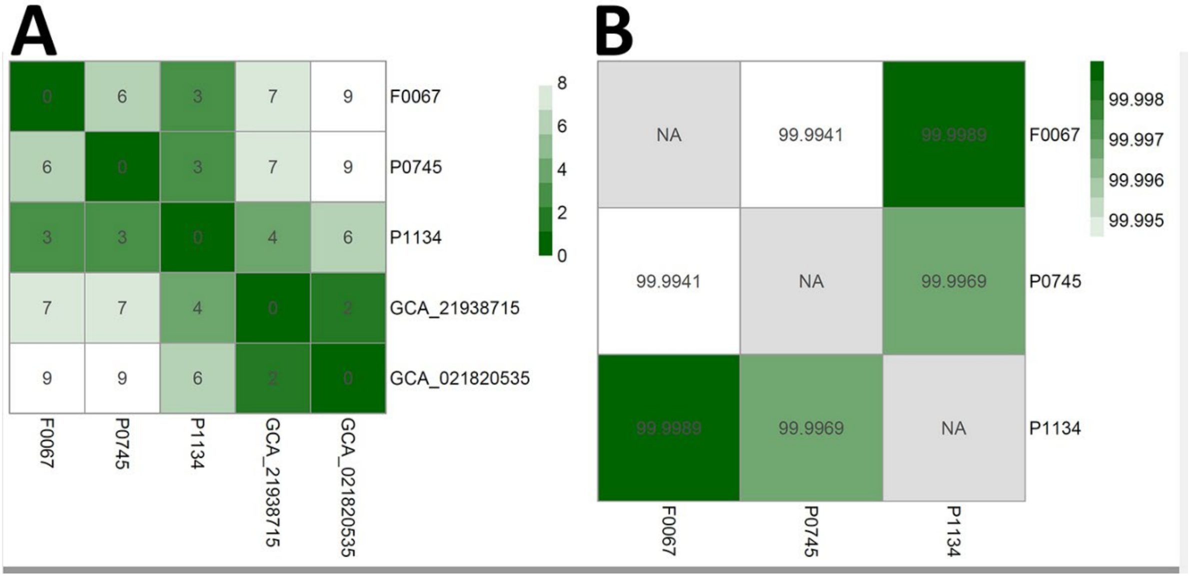

Appendix Figure 2. A) Heatmap showing the allelic differences from the cgMLST analysis of the Swiss isolates involved in the mixed cluster together with genomes GCA_21938715 and GCA_021820535 from the University Hospital Jena, Germany. B) Heatmap illustrating FastANI similarity results among the Swiss isolates involved in the mixed cluster.

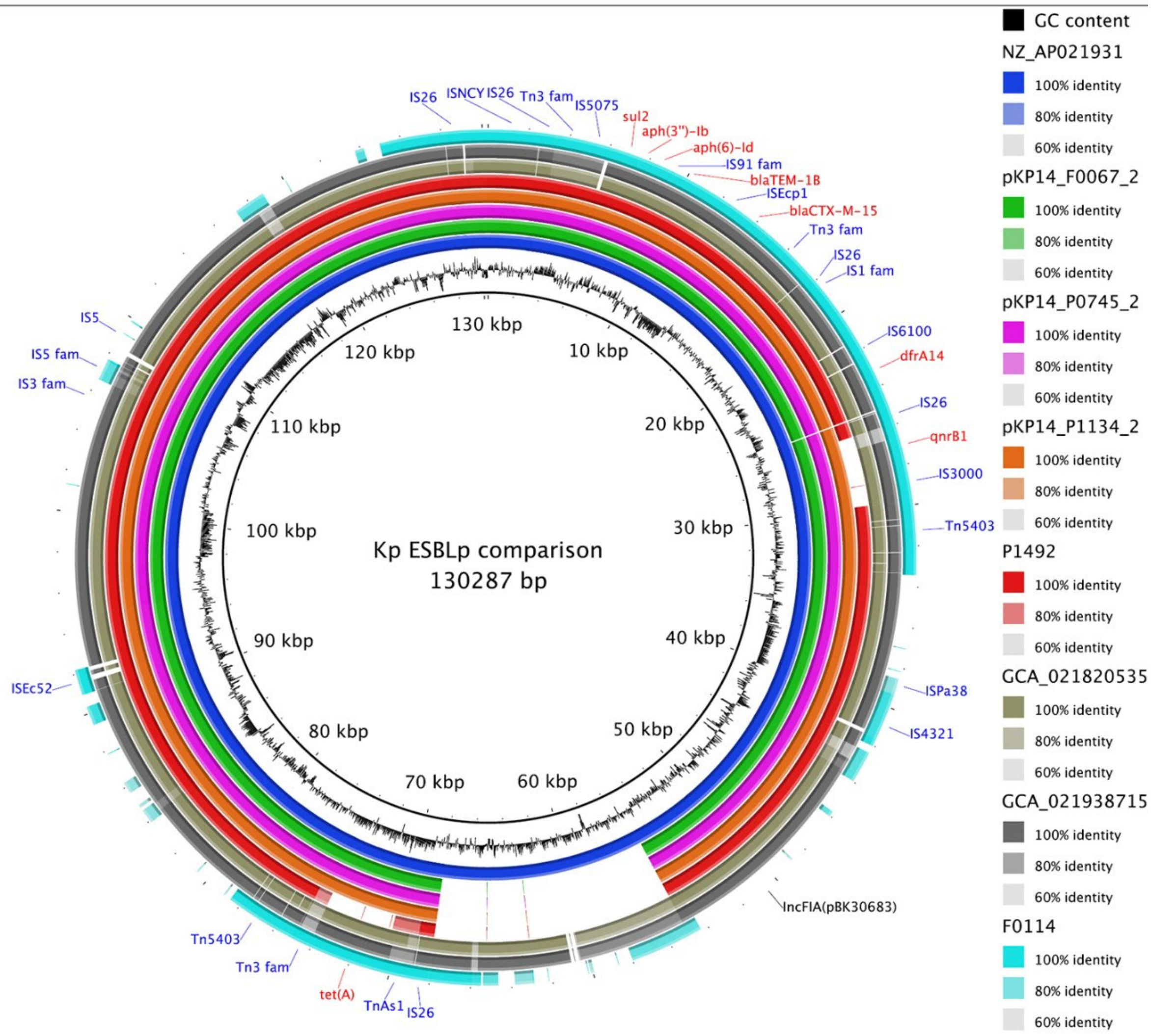

Appendix Figure 3. BRIG comparison of the metal resistance plasmid detected among all three cluster isolates. Green ring: plasmid of the food ESBL-K. pneumoniae isolate (F0067). Pink ring: plasmid of ESBL-K. pneumoniae isolate from patient A (P0745). Orange ring: plasmid of the ESBL-K. pneumoniae isolate of patient B (P1134). The color intensity of the concentric rings represents the percentage of identity compared to the reference used (plasmid of F0067). GC content and plasmid length are displayed as inner rings. Selected metal tolerance/resistance genes, replicons, and insertion sequences (IS) and transposons (Tnp) are marked in red, black, and blue, respectively.
